# Prevalence of HIV related oral lesions in people living with HIV and on combined antiretroviral therapy: a Nigerian experience

**DOI:** 10.11604/pamj.2018.31.180.13574

**Published:** 2018-11-14

**Authors:** Eweka Olutola Mary, Ogbenna Ann Abiola, Gbajabiamila Titilola, Ogundana Oladunni Mojirayo, Akanmu Alani Sulaimon

**Affiliations:** 1 Oral Medicine Unit, Department of Preventive Dentistry, College of Medicine University of Lagos, Lagos, Nigeria; 2 Department of Haematology and Blood Transfusion, College of Medicine University of Lagos, Lagos, Nigeria; 3 Nigerian Institute of Medical Research (NIMR), Lagos, Nigeria; 4 Department of Oral and Maxillofacial Pathology/Biology, College of Medicine University of Lagos, Lagos, Nigeria

**Keywords:** Oral lesions, cART, HIV

## Abstract

**Introduction:**

oral lesions comprise significant clinical features of HIV infection and are often indicators of immune suppression. However, the advent of antiretroviral therapy has significantly reduced its prevalence. The aim of this study was to relate the prevalence of oral lesions of HIV to treatment outcome of Combined Antiretroviral Therapy (cART) in a Nigerian HIV adult population.

**Methods:**

a cross- sectional study was conducted on 491 People Living with HIV (PLWHIV) on cART from two HIV centres in Lagos state, Nigeria. The EC-clearing house guidelines were employed to categorise oral lesions. Presence or absence of these lesions was reconciled with CD4+ cell count as a measure of efficacy of cART treatment.

**Results:**

a total of 491 PLWHIV on cART were enrolled, 366 (74.5%) were females and 125 (25.5%) were males. Age ranged between 18-80 years, with a mean of 41.2 ± 9.1 years. On examination, 12 (2.4%) patients presented with HIV oral lesions. Oral hyperpigmentation (10, 2.0%) was the most common lesion seen, followed by oral ulcers (2,0.4%). Majority (75%) of the affected patients were on a Lamivudine containing regimen. 7 out of the 12 patients with oral lesions had CD4+ cell count between 200-500 cell/mm^3^ prior to cART initiation. Eleven (92%) of the patients with oral lesions had significant improvement of their CD4+ cell count after cART administration.

**Conclusion:**

the prevalence of oral lesions in HIV patients on cART therapy in Lagos is low. Oral hyperpigmentation and oral ulcers are the most frequent lesions seen. The presence or absence of oral lesions were not associated with CD4+ cell count. Therefore, we conclude that the oral lesions seen in HIV patients on cART may not be a direct manifestation of the disease.

## Introduction

Oral lesions form significant early clinical features of HIV infection [1]. These lesions are often indicators of immune suppression and can be used for early testing, diagnosis and management of patients with HIV/ AIDS. Oral lesions therefore contribute largely to patients' morbidity, affecting the psychological and economic functioning of the individual and community [2]. They may be classified into infections such as fungal, viral and bacterial infections, neoplasms such as Kaposi's sarcoma and non-specific presentations such as aphthous ulcerations and salivary gland diseases [3, 4]. The overall prevalence of oral lesions in HIV infected patients has changed since the advent of combination Anti-Retroviral Therapy (cART). For instance, several studies have shown considerable reduction in prevalence of herpes labialis and periodontal diseases along with other oral lesions from 80% to about 30% after the institution of cART [5] and in HIV-associated opportunistic infections [6, 7]. Oral candidiasis (OC) has been shown to be the most common oral lesion seen in HIV infected patients, however with the advent of cART, most studies reported a decline in its occurrence. In a study of 93 patients, 7% of patients on protease inhibitors (PI) had oral candidiasis, compared with 36% in non-PI treated patients [8]. Schmidt-Westhausen *et al.* (2000) detected OC in 10 out of 103 (9.7%) of their study subjects who had been on cART for 4 weeks and in none after 6 months of therapy (N=61) [9]. Unlike most other oral manifestations of HIV, which decrease with use of cART, studies from the USA and the United Kingdom (UK) have described an increase in the prevalence of oral warts with cART [10-12], which may reach statistical significance.

Other lesions that are showing a trend of rising prevalence include HIV- related salivary gland disease [10]. The goal of cART should be maximal and durable viral suppression, restoration and preservation of the immune system with resultant resolution of opportunistic illnesses and improvement in the quality of life through ease of use of the regimen with minimal side-effects to enhance adherence. This should translate to a reduction of HIV-related morbidity including oral manifestations. Reduction of viral load will prevent progressive immunodeficiency, decrease the risk of the emergence of resistant viruses and decrease the risk of viral transmission [13]. The potent combination therapies have proven effective in suppressing plasma-HIV viral load below detectable limits and elevating CD4+ lymphocyte cell counts. Consequently, the immune status for the therapy adherent patients improves significantly. However, some patients fail to achieve complete viral suppression [14, 15]. It has been shown in various studies that the prevalence of HIV-related oral lesions reduces significantly with cART. The reported percentage decrease varied from 10% in a USA study on 570 patients [10] to 50% in a Mexican study on selected 1000 HIV patients over a period of 12 years [16]. In a Nigerian study about 80% of the lesions cleared with use of cART [7]. However, cART sometimes achieves suboptimal results with less than fifty percent of patients achieving therapeutic goal. This is due to a variety of reasons such as medication intolerance/ side effects, prior ineffective antiretroviral therapy and infection with a drug-resistant strain of HIV. However, non-adherence with antiretroviral therapy is the major reason most individuals fail to benefit from cART [17]. Nearly all the reported studies had been conducted in industrialised countries and literatures concerning the behaviour of HIV related oral lesions in patients undergoing cART is scarce. This study therefore seeks to determine the prevalence of HIV related oral lesion on patients undergoing cART therapy and to assess the therapeutic effects of cART on the clinical presentations of these lesions and its relation to CD4+ cell count in a Nigerian adult population.

## Methods

A cross-sectional study was conducted in 491 HIV infected patients on cART therapy, who attended the HIV Clinics of the Lagos University Teaching Hospital (LUTH) and the Nigerian Institute of Medical Research (NIMR). The study was conducted over a period of 5 months and the patients were assessed for presence or absence of HIV-related oral lesions conforming to EC-Clearinghouse, 1993 guidelines on the diagnostic criteria for classifying oral lesions in HIV [18]. Data were collected using a structured interviewer administered questionnaire which included their demographic data, information regarding the time of testing for HIV, source of the disease, if they noticed any previous oral or facial lesions or conditions prior to presentation, how they treated these conditions, if there were improvement with local treatment. History of use of cART were also obtained from the patients, for example- duration of use, names of the specific drugs, reasons for change if any, use if consistent or haphazard, and improvement after therapy. Clinical examination was done with the aid of sterile dental instruments under bright light source. Information obtained included; oral hygiene status, presence of HIV related oral lesions and other lesions like dental caries, tooth discoloration and so on. Laboratory investigations included; HIV screening and CD4+ cell count which were obtained at start of cART and latest test result at the time of examination for the study documented.

Participants were grouped into 3 based on the CDC immunologic classification (category) for HIV infection; those with CD4+ cell count ≥ 500/mm^3^ were classified as group I, those with CD4+ cell count between 200/mm^3^ and 500/mm^3^ were classified as group II and those with CD4 + cell count < 200/mm^3^ were classified as group III. Viral load results were not obtained for majority of the patients due to challenges related to provision of the test. Information regarding treatment of patients with cART was also obtained - name of medications, duration of use and previous medications used were all documented. Ethical approval was given by the Ethics and Research Committee of the Lagos University Teaching Hospital, along with permission from the Deputy Director of Research NIMR. After full explanation of the study to patients; those who declined were excluded while those who gave consent were recruited into the study. Both verbal and written consent were obtained from all the respondents. Privacy and confidentiality were managed by examining the recruited patients in private as well as prior coding of administered questionnaires. Data were analysed using the software SPSS for windows (version 21: SPSS Chicago, IL). Categorical data were presented as numbers and percentages and continuous data as mean ± standard deviation; where data was parametric and median values (interquartile range) utilized for non-parametric data. Chi square test was used to assess the relationship between categorical variables. Fisher's exact test was used for 2 × 2 tables or where the requirements for test could not be met. Paired T test applied to compare mean CD4 cell count at initiation of cART and on examination at recruitment. The 5% significance level was used.

## Results

A total of 491 people living with HIV on cART were enrolled into the study. Participants age range between 18-80 years, with a mean of 41.2 ± 9.1 years. There were 367(74.7%) females and 124(25.3%) males with a female to male ratio of 3:1. Most of the participants were married (66.9%), and up to 44.0% of them had at least secondary school education. Igbo ethnic group represented the most prevalent group (191, 38.9%) followed closely by Yoruba ethnic group (168, 34.2%). Majority 439(89.4%) of the PLWHIV in this study claim to be Christians, while very few of them (11.2%) consume alcohol and cigarette smoking habit was very rare (2.9%) Table 1. Seventy-two (14.7%) of the respondents gave a history of presence of oral lesions suggestive of HIV infection prior to commencement of cART, while 85.3% had never experienced oral lesions suggestive of HIV. Of those who gave a history of oral lesions, 28(38.9%) claimed these lesions cleared after the administration of cART alone, while 21(29.2%) acknowledged the use of other medications along with cART to treat the lesions. The median duration of oral lesions was 9 ±(1-36) months and recurrence was reported in 18(26%) of the respondents (Table 2). Distributions of oral lesions are shown in Table 2. Majority 426 of the total number of patients in this study had been diagnosed of HIV for over a year while the remainder 65 were diagnosed within one year. Only 12(2.4%) of the participants had observable oral lesions suggestive of HIV, and 10 of these lesions were hyperpigmentation of the oral mucosa primarily affecting the tongue and the buccal mucosa. The other patients had oral ulcers suggestive of aphthous ulcerations (Table 3). A total of six out of the 10 patients with oral hyperpigmentation were on regimen containing Lamivudine (75%), this was followed by regimen containing Tenofovir 5(62.5%).

**Table 1:** demographic data of HIV patients on HAART

	Frequency	Percentage (%)
**Demographic data**		
Age (mean in years)	41.22 ± 9.06	
	Range: 18-80	
**Sex**		
Male	125	25.4
Female	366	74.4
**Marital status**		
Single	96	19.6
Married	328	66.8
Divorced	25	5.1
Widowed	41	8.4
**Level of education**		
None	6	1.2
Primary	102	20.8
Secondary	215	43.8
Tertiary	168	34.2
**Religion**		
Christianity	459	89.4
Islam	51	10.4
Others	1	0.2
**Ethnic group**		
Yoruba	167	34.0
Igbo	191	38.9
Hausa	10	2.0
Ijaw/Edo	34	6.9
Efik	28	5.7
Others	61	12.4
**Bio demographic data**		
Cigarette smoking		
Yes *	14	2.9
No	477	97.1
**Alcohol consumption**		
Yes*	57	11.2
No	451	88.8
Route of infection **		
Sexual route	44	9.0
Single partner	62	12.6
Multiple partner	15	3.1
Same sex partner	7	1.4
Sharing sterile instruments	28	6.1
Blood transfusion	21	4.5
Unknown	302	69.9
**no of years since diagnosis**		
<1year	63	12.8
> 0r=1 year	428	87.2
Total	491	100.0

**Table 2:** proportion of patients with history of oral lesions suggestive of HIV prior to presentation

	Frequency	Percentage
**Oral lesions**		
No lesions ever present	419	85.3
Patients with previous lesion	72	14.7
**Features of oral lesions**		
Wound /Sores /Ulcers	22	4.3
White patch/Thrush	16	3.1
Growth/Swelling	10	2.0
Bleeding	6	1.2
Rashes	6	1.2
Hyperpigmented/black patches	3	0.6
Others	9	1.8
Total	72	14.7
**Did lesions clear with ARV**		
Yes	28	40.0
No	25	35.7
I don’t know	19	24.3
Total	72	100
**Did you have to treat with other medications?**		
Yes	21	30.0
No	34	48.6
I don’t know	17	21.4
Total	72	100
**Did the medication/s clear the lesion/s?**		
Yes	16	22.9
No	10	14.3
I don’t know	46	62.8
Total	72	100
**Did lesions ever reoccur?**		
Yes	18	25.7
No	23	32.9
I don’t know	31	41.4
Total	72	100

**Table 3:** clinical presentation of oral lesions of HIV observed during oral examination and medications used

Clinical presentation and medications used	Frequency	Percentage(%)
**Presence of oral lesion**		
Yes	12	2.4
No	479	97.6
Total	491	100
**Types of lesions**		
Hyperpigmentation	10	2.0
Ulcers	2	0.4
Total lesions seen	12	2.4
**Medications used**		
Regimen containing zidovudine	3	37.5
Regimen containing Tenofovir	5	62.5
Regimen containing Efaverenz	3	37.5
Regimen containing Lamivudine	6	75
Regimen containing Emtritabine	2	25
Regimen containing Nevirapine	5	62.5

Initial CD4+ cell count results were obtained for all patients enrolled into the study to access their level of immunosuppression at diagnosis prior to cART initiation. The initial CD4+ cell count results were available for 459 of the enrolled patients; 199(43.4%) had a CD4+ cell count less than 200/mm^3^ and 194(42.3%) had a CD4+ cell count between 200-500/mm^3^. Frequencies of oral lesions in these patients are shown in Table 4. In this study, only 2 out of the 12 patients with oral lesions suggestive of HIV reported previous episodes of such lesions prior to commencing cART, one was a case of oral ulcer and the other a case of oral hyperpigmentation. Five of the patients with oral lesions had a CD4+ cell count less than 200cell/mm^3^ (group 1), while the other 7 were in group 2 (CD4+ cell count between 200-500 cell/mm3) prior to cART initiation. With the definition of significant immunological response in HIV as a rise in CD+ cell count by100 cells/mm3 after more than 6 months of therapy; 11 of the 12 patients with oral lesions had significant increases in the cell count after 6 months of cART therapy (Table 5). Of the 12 patients who presented with oral lesions of HIV at examination, only 1 had a previous history of oral lesion prior to initiation of cART. Other patients did not have any lesion suggestive of HIV prior to cART initiation. Mean CD4 count in cells/mm3 of these patients was 204.5 (+ 107.7) at initiation of cART and 468.7 (+ 18.5) at presentation, with a median duration of 38.5 (22.5-93.8) months. This showed an improvement in their immune status, yet there was an increase in incidence of oral lesions (p=0.002). There was no statistically significant association between the presence of oral lesions and history of cigarette smoking.

**Table 4:** CD4 Classification of all patients with and without history of oral lesions suggestive of HIV

CD4	No oral lesions	Sores/ ulcers/ wounds	White patch / candidiasis	Growth	Bleeding	Rashes	Hyperpigmentation	Others	Total
>500	56(85%)	3(4.5%)	1(1.5%)	0 (0%)	1(1.5%)	2(3.0%)	1(1.5%)	2 (3%)	66(14.3%)
200-500	169 (87.1%)	8(4.1%)	3(1.5%)	7(3.6%)	3(1.5%)	0(0%)	0(0%)	4 (2.2%)	194(42.3%)
<199.99	170 (85.5%)	8(4 %)	10(5%)	2(1%)	0(0%)	4(2%)	2(1%)	3(1.5%)	199(43.4%)
Total									459(100%)

**Table 5:** oral lesions in relation to the CD4 count of patients before and after cART initiation

Oral lesions	Duration of cART in months	Previous history of oral lesions prior to cART	CD4 count @ diagnosis	CD4 count @ cART initiation	CD4 count @ examination
Hyperpigmentation	108	nill	178	178	695
Oral ulcer	120	nill	213	191	516
Hyperpigmentation	12	Oral ulcers	295	295	408
Hyperpigmentation	22	nill	455	286	455
Hyperpigmentation	51	nill	270	156	502
Hyperpigmentation	36	nill	239	239	516
Hyperpigmentation	-	Hyperpigmentation	54	54	502
Hyperpigmentation	41	nill	386	386	222
Oral ulcer	-	nill	-	-	-
Hyperpigmentation			34	34	464
Hyperpigmentation	-	nill	-	-	-
Hyperpigmentation	36	nill	226	226	407

## Discussion

Various studies have shown prevalence of HIV-related oral lesions reduced significantly with the use of cART. The reported percentage decrease varied from 10% in a USA study [11], 50% in a Mexican study [16] and 84% in a previous study in Lagos, Nigeria [7]. Studies examining the effect of cART on the prevalence of individual oral manifestations such as oral candidiasis, oral hairy leukoplakia, HIV-related periodontal diseases, Kaposi's sarcoma (KS), oral papilloma, and HIV-related salivary gland disease showed reduction in the prevalence of these lesions [4, 9, 10, 16]. The current study also agrees with these findings, as 40% of study participants with history of oral lesions prior to commencement of cART reported that the lesion resolved with the use of cART. Though seventy-two (14.7%) of respondents had a history of oral lesions suggestive of HIV prior to administration of cART, only 12 (2.4%) of the patients had lesions at recruitment by which time mean duration on cART was 36 months. This may support the previously mentioned studies of the positive effects of cART administration on oral lesions of HIV [7, 10, 19]. Eweka *et al.* (2012) in their study showed a high prevalence of oral lesions among cART naive HIV patients (38.4%) but after 3 months of cART administration 84% of the lesions cleared [7]. This further showed that with improvement of the immune status there will be a resultant improvement in the oral lesions with cART. The predominant oral manifestation was hyperpigmentation, occuring in 10 out of the 12 participants. The process of hyperpigmentation is complex and the specific mechanisms that causes it in the context of HIV infection are unknown. It may be associated with HIV-induced cytokine dysregulation with the medications commonly prescribed to HIV-seropositive persons, and with adrenocortical dysfunction, which is not uncommon in HIV-seropositive subjects with AIDS [20]. Reported prevalence of hyperpigmentation varies from as low as 5.2% in children in Tanzania to as high as 38% in Venezuela [21, 22].

Although it could not be fully ascertained when the oral pigmentation occurred during the course of cART administration, majority of the patients were certain the pigmentation was not present before cART therapy except in one patient who had oral hyperpigmentation prior to cART administration. It was also observed that majority of participants with hyperpigmentation were either on Lamivudine, Tenofovir or nevirapine containing regimen, none of which is known to be associated with hyperpigmentation. The major ARV which has been associated with oral hyperpigmentation is regimen containing zidovudine. [23] Zidovudine has an established adverse drug reaction of hyperpigmentation of the skin and nails [24]. Though antiretroviral drugs could be responsible for some of the oral hyperpigmentation seen in some studies [25, 26], other research findings suggest some other drugs used in treating concomitant associated diseases such as clofazimine and ketoconazole could increase the α-melanocyte stimulating hormone. On the other hand, some researchers could not find any systemic or local cause for the oral hyperpigmentation and have suggested it may be idiopathic [27, 28]. There was no association between the patients who smoked and those with oral lesions particularly oral hyperpigmentation. Aphthous-like ulcers were seen in 2 of our patients; this condition is seen in HIV patients and categorized by the EEC Clearinghouse as group 2 lesions, (lesions seen in HIV). Aphthous-like ulcer may not necessarily be a direct lesion of HIV as it appeared minimal and could occur in many HIV seronegative patients who are prone to ulcerations. The current study also reported 5 of the patients with oral lesions to have a CD4+ cell count less than 200cell/mm^3^, while the other 7 patients had CD4+ cell count between 200-500 cell/mm^3^) prior to cART initiation. There was significant improvement in the CD4+ cell count after cART administration in 11 of the respondents with post cART oral lesions. These patients gave no prior history of oral lesions related to HIV before initiation of HAART. This further supports our suspicion that the hyperpigmentation may not be a direct manifestation of the disease, but perhaps is a side effect of medications used.

## Conclusion

The prevalence of oral lesions in people living with HIV on cART therapy in Lagos is low. Oral hyperpigmentation and oral ulcers are the most frequently observed lesions. As the presence of oral lesions in PLWHIV in this study had no association with their CD4+ cell count; this study therefore infers that oral lesions seen in HIV patients on cART may not be a direct manifestation of the disease.

### What is known about this topic

- It has been known that prevalence of oral lesions in HIV seropositive patients reduce considerably after administration of cART due to improvement of their immunity;
- It is also known that side effects of the antiretroviral medications may manifest as oral lesions within the oral cavity affecting the aesthetics and quality of life of the patients.

### What this study adds

- With the large population of Nigerians living with HIV/AIDS and on cART, little information has been documented on either the positive or negative effects of these medications on the oral lesions. This paper has therefore added to knowledge by confirming that the prevalence of HIV related oral lesions in patients on cART is low. In addition, presence of oral lesions in People living with HIV in Nigeria does not appear to have any connection with cART therapy.

## Competing interests

The authors declare no competing interests.
